# Loss of heterozygosity at 7p in Wilms' tumour development

**DOI:** 10.1054/bjoc.1999.0922

**Published:** 2000-01-18

**Authors:** R M Powlesland, A K Charles, K T A Malik, P A Reynolds, S Pires, M Boavida, K W Brown

**Affiliations:** CLIC Research Unit, University of Bristol, Department of Pathology and Microbiology, School of Medical Sciences, University Walk, Bristol, BS8 1TD, UK; Department of Paediatric Pathology, St Michael's Hospital, Southwell Street, Bristol, BS2 8EG, UK; Centro de Genetica Humana, Instituto Nacional De Saude, Dr Ricardo Jorge, Av. Padre Cruz, Lisboa Codex, 1699, Portugal

**Keywords:** loss of heterozygosity, chromosome 7, Wilms' tumour, tumour suppressor gene

## Abstract

Chromosome 7p alterations have been implicated in the development of Wilms' tumour (WT) by previous studies of tumour cytogenetics, and by our analysis of a constitutional translocation (t(1;7)(q42;p15)) in a child with WT and radial aplasia. We therefore used polymorphic microsatellite markers on 7p for a loss of heterozygosity (LOH) study, and found LOH in seven out of 77 informative WTs (9%). The common region of LOH was 7p15–7p22, which contains the region disrupted by the t(1;7) breakpoint. Four WTs with 7p LOH had other genetic changes; a germline *WT1* mutation with 11p LOH, LOH at 11p, LOH at 16q, and loss of imprinting of *IGF2*. Analysis of three tumour-associated lesions from 7p LOH cases revealed a cystic nephroma-like area also having 7p LOH. However, a nephrogenic rest and a contralateral WT from the two other cases showed no 7p LOH. No particular clinical phenotype was associated with the WTs which showed 7p LOH. The frequency and pattern of 7p LOH demonstrated in our studies indicate the presence of a tumour suppressor gene at 7p involved in the development of Wilms' tumour. © 2000 Cancer Research Campaign


*British Journal of Cancer* (2000) **82**(2), 323–329
© 2000 Cancer Research Campaign
Article no. bjoc.1999.0922

### Loss of heterozygosity at 7p in Wilms' tumour development

**RM Powlesland[1], AK Charles[2], KTA Malik[1], PA Reynolds[1], S Pires[3], M Boavida[3] and KW Brown[1]**

[1]CLIC Research Unit, University of Bristol, Department of Pathology and Microbiology, School of Medical Sciences, University Walk, Bristol BS8 1TD, UK; [2]Department of Paediatric Pathology, St Michael's Hospital, Southwell Street, Bristol BS2 8EG, UK; [3]Centro de Genetica Humana, Instituto Nacional De Saude, Dr Ricardo Jorge, Av. Padre Cruz, 1699 Lisboa Codex, Portugal

**Summary** Chromosome 7p alterations have been implicated in the development of Wilms' tumour (WT) by previous studies of tumour cytogenetics, and by our analysis of a constitutional translocation (t(1;7)(q42;p15)) in a child with WT and radial aplasia. We therefore used polymorphic microsatellite markers on 7p for a loss of heterozygosity (LOH) study, and found LOH in seven out of 77 informative WTs (9%). The common region of LOH was 7p15–7p22, which contains the region disrupted by the t(1;7) breakpoint. Four WTs with 7p LOH had other genetic changes; a germline *WT1* mutation with 11p LOH, LOH at 11p, LOH at 16q, and loss of imprinting of *IGF2*. Analysis of three tumour-associated lesions from 7p LOH cases revealed a cystic nephroma-like area also having 7p LOH. However, a nephrogenic rest and a contralateral WT from the two other cases showed no 7p LOH. No particular clinical phenotype was associated with the WTs which showed 7p LOH. The frequency and pattern of 7p LOH demonstrated in our studies indicate the presence of a tumour suppressor gene at 7p involved in the development of Wilms' tumour. © 2000 Cancer Research Campaign

**Keywords:** loss of heterozygosity; chromosome 7; Wilms' tumour; tumour suppressor gene

Wilms' tumour (WT) is an embryonal renal neoplasm and is one of the commonest solid tumours of childhood, affecting approximately one in 10 000 children (Coppes et al, 1995). It is a genetically complex tumour, with multiple genes involved in familial and sporadic forms (Huff and Saunders, 1993; Hastie, 1994). Only the *WT1* tumour suppressor gene at 11p13 has been cloned, by virtue of its involvement in the WAGR syndrome (**W**ilms' tumour, **A**niridia, **G**enitourinary abnormalities and mental **R**etardation), in which there are chromosomal deletions at 11p13 (Call et al, 1990; Gessler et al, 1990; Huang et al, 1990). Loss of heterozygosity (LOH) studies in sporadic tumours have identified other potential tumour suppressor loci at 11p15 (Ping et al, 1989; Reeve et al, 1989), 16q (Maw et al, 1992), 1p (Grundy et al, 1994), 7p15 (Wilmore et al, 1994) and 11q (Radice et al, 1995). Relaxation of imprinting at 11p15, leading to biallelic expression of the normally imprinted gene *IGF2*, is a common event in sporadic WTs not showing 11p LOH (Ogawa et al, 1993; Rainier et al, 1993). Overexpression of *IGF2* also occurs in the Beckwith–Wiedemann syndrome (BWS), which predisposes to WT, and which is associated with 11p15 partial trisomy, uniparental paternal disomy of 11p15, and imprinting mutations at 11p15 (Reik and Maher, 1997). Two familial loci have recently been identified at 17q and at 19q by linkage analysis (Rahman et al, 1996; McDonald et al, 1998). p53 mutations, 16q LOH and 1p LOH are all associated with a poor prognosis in WT (Grundy et al, 1994; Malkin et al, 1994; Lahoti et al, 1996).

Despite the isolation of *WT1* and the identification of several alternate loci, there are still many WTs in which no genetic abnormalities have been detected. In WTs showing 11p LOH and/or *WT1* mutations, premalignant lesions associated with the tumours (nephrogenic rests (Beckwith et al, 1990)) have been shown to contain identical LOH or mutations (Park et al, 1993; Charles et al, 1998*a*), implying that further genetic changes are required for progress to malignancy. Thus in many WTs the initiating genetic events are still unclear, as are the genetic pathways by which nephrogenic rests progress to malignancy.

The discovery of cytogenetic abnormalities in syndromes predisposing to WT, i.e. 11p13 deletions in WAGR syndrome (Franke et al, 1979), 11p15 partial trisomy and translocations in BWS (Reik and Maher, 1997), have proved critical for the identification of WT genes. We therefore became interested in a patient who presented with WT, a nephrogenic rest in the contralateral kidney and an unusual set of congenital abnormalities (bilateral radial aplasia and other skeletal abnormalities) not previously associated with WT (Hewitt et al, 1991). This patient had a constitutional balanced chromosome translocation; t(1;7) (q42;p15) (Hewitt et al, 1991). In the patient's WT, both translocated chromosomes were retained, but an isochromosome 7q was formed, making the tumour monosomic for 7p and trisomic for 7q (confirmed by molecular studies) (Wilmore et al, 1994). The bilateral nature of this patient's kidney disease, the molecular and cytogenetic findings, together with 7p LOH in another sporadic WT, led us to propose that chromosome 7p15 contains a tumour suppressor gene involved in WT development (Wilmore et al, 1994).

We have now accurately mapped the breakpoint on 7p to a 500 bp region, by fluorescence in situ hybridization (FISH) analysis with human YAC and cosmid clones, and by higher resolution molecular studies (Reynolds et al, 1996 and unpublished observations). In order to investigate the potential involvement of this locus in other WTs, we have used a panel of polymorphic

*Received 16 April 1999*
*Revised 22 July 1999*
*Accepted 25 August 1999*

*Correspondence to:* KW Brown

**Table 1** PCR primers used for LOH studies

| Location | Marker | Repeat type | Primer sequences (5′→3′) | Product size (bp) | Reference |
|---|---|---|---|---|---|
| 7p22 | D7S517 | D | F: tggagaagccatgtgagt<br>R: agctgtaattagttgctggtttga | 250 | Dib et al, 1996 |
| 7p15 | D7S795 | T | F: taaatgcctggagtctggag<br>R: tcacagaaacccaatccact | 230 | Sheffield et al, 1995 |
| 7p15 | D7S2211 | T | F: gataggtgcagcaaaccact<br>R: ctgccctcacttggacttaa | 380 | Sheffield et al, 1995 |
| 7p15 | D7S3023 (MS0003) | D | F: tcaccccaggagatcagttgttgg<br>R: cagcaatgccaagattcaaaccag | 240 | Keen et al, 1995 |
| 7p15 | SICA | D | F: aggcctggttccttactcag<br>R: aaggcatgcaaaatggaactgc | 110 | Unpublished |
| 7p15 | D7S683 | D | F: ttttaggcttatcaaacggg<br>R: ggtgtctgtctacatccaactg | 260 | Dib et al, 1996 |
| 7p13 | D7S691 | D | F: gggtgattaatgcttgctta<br>R: gcttgattttccaacagg | 130 | Dib et al, 1996 |
| 7q22 | D7S554 | D | F: gtctaattacccacatttccct<br>R: gttccatatttaaaagactcagtga | 250 | Dib et al, 1996 |
| 11p15 | TH | T | F: gggtatctgggctctggggt<br>R: ggtcacagggaacacagactcc | 110 | Hearne et al, 1992 |
| 11p15 | D11S1999 | T | F: tacatggcagcaggcatata<br>R: gagtaaacaagattgctagataggc | 120 | Sheffield et al, 1995 |
| 11p13 | D11S1392 | T | F: ttgcatccatacggaaagtc<br>R: acatctgagacttgtagtagaaggc | 200 | Sheffield et al, 1995 |
| 16q21 | D16S265 | D | F: ccagacatggcagtctcta<br>R: agtcctctgtgcactttgt | 100 | Weber et al, 1990 |

D = dinucleotide repeat, T = tetranucleotide repeat, F = forward primer, R = reverse primer. Physical mapping data from Chumakov et al (1995).

microsatellite markers (including one novel CA repeat close to the breakpoint), to determine the frequency of LOH in this region. This is the largest study of 7p LOH in WT, and we show that LOH at 7p occurs in 9% of WTs, and that the common region lost includes the area disrupted by the t(1;7) translocation.

### MATERIALS AND METHODS

### Tissues and DNA and RNA extraction

Sixty-one WTs and corresponding normal kidney samples were collected in Bristol. One patent had WAGR syndrome, one BWS, two were first cousins and one patient had a constitutional t(1;7) with skeletal abnormalities (this is patient WT21 in Figure 1) (Hewitt et al, 1991). All other cases were sporadic with no obvious genetic predisposition. Eighteen WTs were from Portugal, and clinical data were not available for these.

Fresh normal kidney and tumour tissues were snap-frozen in liquid nitrogen and then stored at –70°C. DNA and RNA were extracted from frozen tissues as described previously (Maitland et al, 1987). Where fresh tissue was not available, or where the lesion was too small to be dissected from the gross tumour tissue, DNA was extracted using microdissection from paraffin sections (Charles et al, 1998*a*).

### Polymerase chain reaction for LOH

The primers used for polymerase chain reaction (PCR) are shown in Table 1. One microlitre of purified DNA (0.5 μg $\mu l^{-1}$) or 5 μl of microdissection supernatant were used for each PCR reaction in a final volume of 25 μl, overlaid with 50 μl mineral oil. The mix consisted of DNA (as above) in 10 mM Tris–HCl pH 9.0, 1.5 mM magnesium chloride, 50 mM potassium chloride, 0.1% Triton X-100, 0.01% gelatin, 1 μM each of forward and reverse primer and 0.25 units Supertaq polymerase (HT Biotechnology Ltd, Cambridge, UK). Tubes were denatured for 3 min at 94°C, followed by 40 cycles of 94°C for 1 min, 55°C for 1 min and 72°C for 1 min. PCR products were run on 6 or 8% non-denaturing polyacrylamide gels and visualized by ethidium bromide staining (Mellersh and Sampson, 1993). All PCRs were set up using aerosol-resistant tips, in a separate room away from where products were analysed. Negative controls were included in each reaction set, and results were repeated if contamination was detected in these.

### Loss of imprinting

Loss of imprinting leading to biallelic expression of *IGF2* was assessed using transcribed polymorphisms in *IGF2* RNA, as described previously (Brown et al, 1996).

### Cytogenetic analysis and Southern blotting

These were carried out using standard methods, as described previously (Wilmore et al, 1994). A minimum of ten metaphases were analysed per tumour and the abnormalities shown in Figure 1 were found in all cells examined.

### RESULTS

### LOH and other genetic changes at 7p

A total of 79 Wilms' tumours were analysed with a panel of six polymorphic microsatellite markers on 7p, and 77 (97%) were informative for at least one of these markers. The markers used were previously mapped dinucleotide and tetranucleotide

 © 2000 Cancer Research Campaign

| Location | Marker | WT21 | WT30 | WT40 | WT42 | WT57 | WT59 | WT73 |
|---|---|---|---|---|---|---|---|---|
| 7p22 | D7S517 | ■ | ■ | ■ | ■ | □ | □ | □ |
| *7p21* | *D7S507* | | | | | | | |
| *7p15* | *D7S503* | | | | | | | |
| *7p15* | *D7S526* | | | | | | | |
| 7p15 | D7S795 | ■ | □ | ■ | ■ | ■ | ■ | ■ |
| | D7S2211 | | ■ | □ | | ■ | | |
| | D7S3023 | ■ | ■ | □ | ■ | □ | □ | ■ |
| | SICA | □ | ■ | ■ | ■ | □ | ■ | □ |
| | D7S683 | □ | □ | | □ | □ | □ | |
| 7p13 | D7S691 | □ | ■ | ☒ | □ | ■ | ■ | ■ |
| *7p12* | *D7S506* | | | | | | | |
| 7q22 | D7S554 | ☒ | ☒ | □ | ☒ | ☒ | □ | □ |
| 11p15 | TH | ☒ | □ | □ | ☒ | ☒ | ☒ | ☒ |
| | D11S1999 | | ■ | ■ | | | | |
| 11p13 | D11S1392 | ☒ | ■ | □ | ☒ | ☒ | ☒ | ☒ |
| | Homozygous WT1 mutation | ☒ | | ■ | | | | |
| 16q21 | D16S265 | ☒ | □ | ☒ | ☒ | ☒ | □ | ■ |
| 11p15 LOI | IGF2 | | □ | □ | ■ | | | |
| | Karyotype | 46,XY, t(1:7)(q42;p15)c, i(7)(q10) | | 46,XX | | 52,XX, +del(1)(p13), i(7)(q10), add(11)(p15), +2,+12,+13, +10,+mar | 46,XY, add(1)(q1), add(3)(p13), −7,–16, +mar,+mar | 45,XX, i(7)(q10), del(16)(q22), −22 |

**Figure 1** Loss of heterozygosity at 7p in Wilms' tumour. Loss of heterozygosity (LOH) was assessed using microsatellite markers on chromosomes 7, 11 and 16. Representative results of LOH studies are shown in Figures 2 and 3. Loss of imprinting (LOI) was assessed using a transcribed polymorphism in *IGF2*. Karyotypes were determined by standard cytogenetic methods; no cells grew from WT30 and WT42. The common region of LOH on 7p was between D7S683 and D7S517 (7p15 to 7p22). 7p markers shown in italics were not used in this work but are mentioned in the discussion. LOH or LOI: ■; no LOH or LOI: ☒; non-informative: □; not done: (blank)

microsatellites, with the exception of S1CA, which is a novel CA repeat that we have isolated from a YAC clone that spans the t(1:7) translocation (Reynolds et al, 1996). This polymorphic CA repeat (observed heterozygosity 58%) maps between D7S683 and D7S3023, to within 500 bp of the translocation breakpoint (Malik and Reynolds, unpublished).

Seven of the 77 tumours (9%) showed LOH at 7p; these results are summarized in Figure 1 and representative gels are shown in Figures 2 and 3. The common region of LOH was between D7S683 (7p15) and D7S517 (7p22), which covers the area containing the t(1;7) breakpoint.

In addition we examined 58 WTs for homozygous deletions at S1CA. All were positive for this marker, demonstrating that there were no homozygous deletions in the region of the t(1;7) breakpoint. Sixteen Wilms' tumour DNAs have also been assessed by Southern blotting for rearrangements using single-copy probes derived from a cosmid clone that spans the t(1:7) translocation, but no gross alterations were apparent within approximately 20 kb of the breakpoint region (data not shown).

### Clinical properties of tumours showing 7p LOH

Clinical data on age of presentation, sex, tumour stage, histology and outcome are given in Table 2 for each of the tumours showing 7p LOH. A comparison of the clinical data for tumours with or without 7p LOH is shown in Table 3 (clinical data were not available for all patients, see Materials and Methods). There did not appear to be any striking differences in clinical features between the two groups; the relapse rate was higher in 7p LOH tumours (29% compared to 12%), but this difference was not statistically significant.

### Other genetic changes and cytogenetics

Cytogenetic analysis was successful in five out of the seven tumours that had 7p LOH (Figure 1). Three of these (WT21, 57 and 73) had an isochromosome 7q, and one (WT59) showed complete loss of one entire chromosome 7, all of which are entirely consistent with the 7p LOH detected in these tumours (Figure 1).

*© 2000 Cancer Research Campaign* 

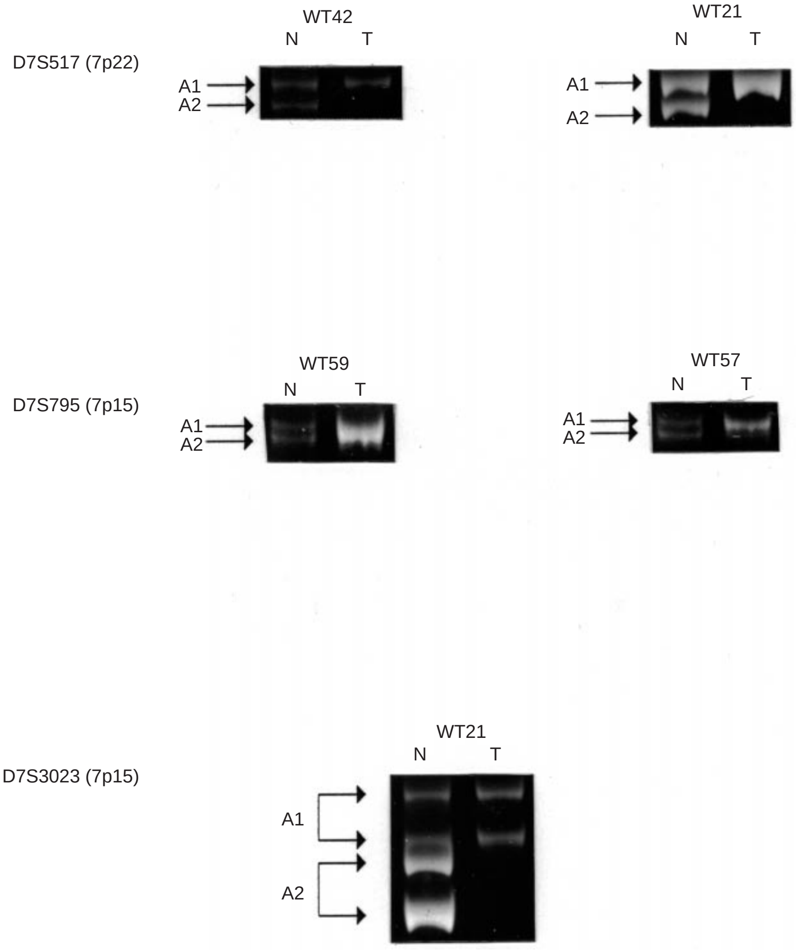

**Figure 2** Examples of 7p LOH in Wilms' tumour. Five examples of LOH at 7p are shown. DNA from normal kidney (N) or Wilms' tumour (T) was amplified by PCR with primers for various microsatellite markers on 7p and the products were resolved on non-denaturing polyacrylamide gels. The tumours analysed are shown above the relevant lanes and the microsatellites are indicated on the left. A1 = larger allele, A2 = smaller allele. With D7S3023, stutter bands produce two bands for each allele (both arrowed)

**Table 2** Clinical data on Wilms' tumours showing LOH at 7p

| Tumour | Sex | Age at diagnosis (months) | Stage | Outcome | Histology |
|---|---|---|---|---|---|
| WT21 | M | 65 | I | A | T[b] |
| WT30 | M | 42 | II | A | T, Rh |
| WT40 | F | 13 | V | D | S, Rh[b] |
| WT42 | M | 40 | II | A | B |
| WT57 | F | 48 | III | R[a] | T[b] |
| WT59 | M | 10 | V | A | B, Rh |
| WT73 | F | 82 | III | R | B |

M = male, F = female, A = alive, D = dead, R = relapsed, T = triphasic, S = stromal predominant, B = blastemal predominant, Rh = rhabdomyoblasts present. [a]Also developed therapy-induced acute myeloid leukaemia. [b]Histology was of post-chemotherapy tumour.

Two patients with 7p LOH (WT30 and WT40) also showed LOH at 11p, which in WT40 was associated with a homozygous *WT1* mutation (Figures 1 and 3). There was a lower frequency of LOH at both 11p13 and 11p15 in the 7p LOH group when compared with those WTs without 7p LOH (Table 3), but these differences were not statistically significant. Both groups showed similar levels of LOH at 16q (Table 3). One tumour with 7p LOH (WT42) showed loss of imprinting at *IGF2* (Figure 1).

### Tumour progression and 7p LOH

In order to determine at which stage of tumour development 7p changes occurred, we investigated LOH in other lesions associated with the patients' WTs.

The patient with WT40 had bilateral tumours; the data in Figure 1 came from the WT initially resected. DNA was subsequently extracted by microdissection from the contralateral tumour and amplified with primers for one informative microsatellite. The second tumour did not show LOH at S1CA (7p15) but clearly did show LOH at D11S1999 (11p15), whereas the original tumour showed LOH at both 7p13 and 11p15 (Figure 3).

In WT57 the tumour contained a cystic nephroma-like area, and it could be clearly seen that the cystic nephroma-like area also had 7p LOH (Figure 3).

WT21 also had associated rests but unfortunately all PCR reactions were unsuccessful in that case. In WT59 there were perilobar nephrogenic rests and one of these was microdissected to produce

*© 2000 Cancer Research Campaign*

**Table 3** Comparison of clinical data and other LOH data for 7p LOH and non-LOH Wilms' tumours

| 7 p LOH | Number of cases | Sex (no.) (%) Male | Female | Mean age at diagnosis (months) | Stage distribution (no.) (%) I | II | III | IV | V | Outcome (no.) (%) Relapsed | Died | Other LOH (no. with LOH/no. informative) (%) 11p13 | 11p15 | 16q |
|---|---|---|---|---|---|---|---|---|---|---|---|---|---|---|
| No | 52 | 22 | 30 | 42.6 | 12 | 16 | 10 | 8 | 6 | 6 | 9 | 13/28 | 20/44 | 6/36 |
| | | 42% | 58% | | 23% | 31% | 19% | 15% | 12% | 12% | 17% | 46% | 45% | 17% |
| Yes | 7 | 4 | 3 | 42.9 | 1 | 2 | 2 | 0 | 2 | 2 | 1 | 2/6[a] | 2/7 | 1/5 |
| | | 57% | 43% | | 14% | 29% | 29% | 0% | 29% | 29% | 14% | 33% | 29% | 20% |

[a]Includes tumour with homozygous *WT1* mutation (WT40).

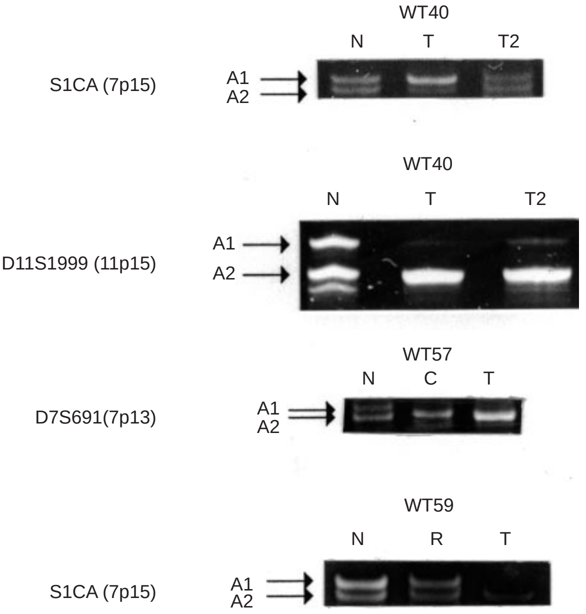

**Figure 3** LOH studies in other lesions associated with Wilms' tumour. DNA was extracted from normal kidney (N) or Wilms' tumour (T) or from microdissections of associated lesions: a second WT (T2; WT40), an area of cystic nephroma (C; WT57), or a perilobar nephrogenic rest (R; WT59). Other details as in Figure 1. In WT40 LOH occurred at 7p15 only in the first tumour (T), not in the second (T2), but both had LOH at 11p15. In WT57 both the cystic nephroma area (C) and the Wilms' tumour (T) showed LOH at 7p15. In WT59 the tumour (T) had LOH at 7p, whereas the nephrogenic rest (R) showed no loss

DNA. Unlike the tumour, the nephrogenic rest did not show LOH at any of these 7p markers (S1CA; Figure 3, D7S795 and D7S691 not shown).

### DISCUSSION

The results described in this paper demonstrate that in a large series, 9% of Wilms' tumours have loss of heterozygosity at 7p, and that the common region of LOH includes the area involved in the constitutional t(1;7) translocation which we have previously mapped (Reynolds et al, 1996). This strongly suggests that there is a novel tumour suppressor gene at 7p15 that is involved in the development of a significant proportion of WTs.

Past and recent work from other laboratories support a role for 7p in WT tumorigenesis. Several cytogenetic studies of sporadic WTs have reported 7p deletions, translocations involving 7p, and isochromosome 7q (Solis et al, 1988; Wang-Wuu et al, 1990; Kaneko et al, 1991; Sawyer et al, 1993; Peier et al, 1995; Riviera, 1995; Fletcher and Renshaw, 1996; Miozzo et al, 1996; Steenman et al, 1997; Lobbert et al, 1998). In some WTs, 7p changes have been found as the sole cytogenetic abnormality (Solis et al, 1988; Wang-Wuu et al, 1990; Sawyer et al, 1993; Peier et al, 1995; Lobbert et al, 1998), implying that these are unlikely to represent random changes. Overall, reviews by several authors have implicated chromosome 7 cytogenetic abnormalities in up to 20% of WTs (Rivera et al, 1985; Wang-Wuu et al, 1990; Kaneko et al, 1991; Austruy et al, 1995).

Our results are the largest study so far of 7p LOH in WT, and our 9% LOH rate is very similar to the 10% (four out of 40) reported recently by Grundy et al (1998), but less than the 27% (three out of 11) found by Miozzo et al (1996). The common region of LOH in our study was between D7S683 and D7S517 (7p15–7p22), which overlaps with the minimal LOH regions identified by Grundy et al (D7S503–D7S517) and Miozzo et al (D7S506–D7S526) (Figure 1) (Miozzo et al, 1996; Grundy et al, 1998). However, the minimal regions defined in these latter reports are separated by approximately 15 cM (Dib et al, 1996), with the common region defined by Grundy et al being distal to that reported by Miozzo et al. In addition, Grundy et al identified a putative homozygous deletion at D7S507 (7p15/p21) in one tumour (Grundy et al, 1998), which lies approximately 25 cM distal to the constitutional t(1;7) breakpoint which we have mapped in our WT patient (Reynolds et al, 1996). Thus it seems likely that there may be two loci on 7p involved in the development of Wilms' tumour; one at 7p15 and another more distal. This would agree with the reported locations of constitutional and somatic translocation breakpoints, which have been identified at both 7p15 (Miozzo et al, 1996; Reynolds et al, 1996) and 7p22 (Rivera, 1995; Lobbert et al, 1998).

We have not identified any particular characteristics associated with 7p LOH (Tables 2 and 3). In contrast, Grundy et al (1998) have suggested that tumours with 7p abnormalities tend to be of early onset and of high stage. Clearly larger numbers of tumours need to be studied, but if there are two WT loci on 7p, then distinct phenotypes may be associated with each locus, complicating the analysis of clinical data when genetic abnormalities have only been defined by LOH.

In four of the WTs with 7p LOH, we have found other associated genetic abnormalities (Figure 1), three of which involve 11p (*WT1* mutation with 11p LOH, 11p LOH and loss of imprinting of *IGF2*). Grundy et al (1998) reported that 11p LOH had been previously identified in one of their WTs with 7p LOH, and others have found chromosome 7 cytogenetic abnormalities in association with a chromosome 11 deletion (Wang-Wuu et al, 1990), and with a germline *WT1* mutation and 11p LOH (Lobbert et al, 1998). Thus

© 2000 Cancer Research Campaign 

it appears that 7p LOH is an event that usually occurs in concert with other genetic abnormalities.

These results suggest that chromosome 7 changes are important in WT development, but that they may be insufficient in themselves for malignant transformation. In order to determine at which stage of WT development 7p LOH was involved, we examined other lesions associated with the tumours (Figure 3). In WT57, a cystic nephroma-like area showed 7p LOH, as did the bulk of the tumour. This supports the contention that cystic nephroma is closely related to Wilms' tumour, and that cystic nephroma-like areas within a WT should be regarded as a part of the tumour, or possibly a precursor lesion (Charles et al, 1998*b*). In contrast, in WT40, where there were bilateral tumours, the second contralateral tumour did not show 7p LOH, whereas both tumours had LOH at 11p15. The first tumour from WT40 had previously been shown to have a homozygous *WT1* mutation (Miyagawa et al, 1998), and we have now shown that the mutation is germline in this patient (data not shown). From the 11p LOH results (Figure 3) we would suggest that the second tumour was also homozygous for the *WT1* mutation. It therefore appears that in the case of WT40, LOH at 7p was a late event that only occurred in one of the bilateral tumours, with the development of homozygosity for a *WT1* mutation being the initiating event in both tumours. In WT59, 7p LOH was found only in the tumour and not in an associated nephrogenic rest (Figure 3). Nephrogenic rests are thought to be premalignant lesions from which malignant Wilms' tumours develop (Beckwith et al, 1990), therefore the WT59 result also suggests that 7p LOH was a relatively late event in the development of that tumour.

Knudson and Strong originally proposed that two rate-limiting events were necessary for WT development, although this did not preclude other non-rate-limiting genetic events being involved (Knudson and Strong, 1972). Park et al have demonstrated that homozygous *WT1* mutations can be found in nephrogenic rests (Park et al, 1993) and our recent studies have confirmed this in two other cases, and shown that in most WTs showing 11p LOH, LOH also occurs in associated nephrogenic rests (Charles et al, 1998*a*). Thus it is clear that whilst *WT1* mutations can initiate WT development, other events may be required for progression to a fully malignant tumour. The results presented in this paper implicate 7p genes in this progression, because some of the tumours with 7p LOH also had 11p alterations, and in others we have found that 7p LOH appears to be a relatively late event.

However, Steenman et al used comparative genomic hybridization to show in one case of WT that deletion of 7p was found in both the tumour and in an associated nephrogenic rest, although loss of 1p was present exclusively in the tumour (Steenman et al, 1997). Together with the germline translocations at 7p (Hewitt et al, 1991; Rivera, 1995), these results suggest that 7p alterations can be initiating events in some WTs. WT development may therefore have a preferred order of genetic changes, but it is the accumulation of events which is critical for the development of malignancy, as observed in more complex multistage cancers (Fearon and Vogelstein, 1990).

### ACKNOWLEDGEMENTS

The authors thank the physicians, surgeons, pathologists and patients and their families for their co-operation, Dr Alan Hedges for the statistical analysis, and the South Western Regional Cytogenetics Service for the tumour karyotypes. This work was funded by the Cancer and Leukaemia in Childhood charity, the National Kidney Research Fund (UK), and the Portuguese Association Against Cancer (NRS/LPCC).

### REFERENCES

Austruy E, Candon S, Henry I, Gyapay G, Tournade MF, Mannens M, Callen D, Junien C and Jeanpierre C (1995) Characterization of regions of chromosomes 12 and 16 involved in nephroblastoma tumorigenesis. *Genes Chromosomes Cancer* **14**: 285–294

Beckwith JB, Kiviat NB and Bonadio JF (1990) Nephrogenic rests, nephroblastomatosis, and the pathogenesis of Wilms' tumor. *Pediatr Pathol* **10**: 1–36

Brown KW, Villar AJ, Bickmore W, ClaytonSmith J, Catchpoole D, Maher ER and Reik W (1996) Imprinting mutation in the Beckwith-Wiedemann syndrome leads to biallelic IGF2 expression through an H19-independent pathway. *Hum Mol Genet* **5**: 2027–2032

Call KM, Glaser T, Ito CY, Buckler AJ, Pelletier J, Haber DA, Rose EA, Kral A, Yeger H and Lewis WH (1990) Isolation and characterization of a zinc finger polypeptide gene at the human chromosome 11 Wilms' tumor locus. *Cell* **60**: 509–520

Charles AK, Brown KW and Berry PJ (1998*a*) Microdissecting the genetic events in nephrogenic rests and Wilms' tumor development. *Am J Pathol* **153**: 991–1000

Charles AK, Vujanic GM and Berry PJ (1998*b*) Renal tumours of childhood. *Histopathology* **32**: 293–309

Chumakov IM, Rigault P, Legall I, Bellannechantelot C, Billault A, Guillou S, Soularue P, Guasconi G, Poullier E, Gros I, Belova M, Sambucy JL, Susini L, Gervy P, Glibert F, Beaufils S, Bui H, Massart C, Detand MF, Dukasz F, Lecoulant S, Ougen P, Perrot V, Saumler M, Soravito C, Bahouayila R, Cohenakenine A, Barillot E, Bertrand S, Codani JJ, Caterina D, Georges I, Lacroix B, Lucotte G, Sahbatou M, Schmit C, Sangouard M, Tubacher E, Dib C, Faure S, Fizames C, Gyapay G, Millasseau P, Nguyen S, Muselet D, Vignal A, Morissette J, Menninger J, Lieman J, Desai T, Banks A, Brayward P, Ward D, Hudson T, Gerety S, Foote S, Stein L, Page DC, Lander ES, Weissenbach J, Lepaslier D and Cohen D (1995) A YAC contig map of the human genome. *Nature* **377**: 175–298.

Coppes MJ, Campbell CE and Williams BRG (1995) *Wilms Tumor: Clinical and Molecular Characterisation*. RG Landes: Austin, Texas

Dib C, Faure S, Fizames C, Samson D, Drouot N, Vignal A, Millasseau P, Marc S, Hazan J, Seboun E, Lathrop M, Gyapay G, Morissette J and Weissenbach J (1996) A comprehensive genetic map of the human genome based on 5,264 microsatellites. *Nature* **380**: 152–154

Fearon ER and Vogelstein B (1990) A genetic model for colorectal tumorigenesis. *Cell* **61**: 759–767

Fletcher JA and Renshaw AA (1996) Isochromosome 7q in adult Wilms' tumor. *Cancer Genet Cytogenet* **86**: 168–169

Franke U, Holmes LB, Atkins L and Riccardi VM (1979) Aniridia Wilms' tumor association: evidence for specific deletion of 11p13. *Cytogenet Cell Genet* **24**: 185–192

Gessler M, Poustka A, Cavenee W, Neve RL, Orkin SH and Bruns GA (1990) Homozygous deletion in Wilms tumours of a zinc-finger gene identified by chromosome jumping. *Nature* **343**: 774–778

Grundy PE, Telzerow PE, Breslow N, Moksness J, Huff V and Paterson MC (1994) Loss of heterozygosity for chromosomes 16Q and 1P in Wilms' tumors predicts an adverse outcome. *Cancer Res* **54**: 2331–2333

Grundy RG, Pritchard J, Scambler P and Cowell JK (1998) Loss of heterozygosity for the short arm of chromosome 7 in sporadic Wilms' tumour. *Oncogene* **17**: 395–400

Hastie ND (1994) The genetics of Wilms' tumor – a case of disrupted development. *Annu Rev Genet* **28**: 523–558

Hearne CM, Ghosh S and Todd JA (1992) Microsatellites for linkage analysis of genetic traits. *Trend Genet* **8**: 288–294

Hewitt M, Lunt PW and Oakhill A (1991) Wilms' tumour and a de novo (1;7) translocation in a child with bilateral radial aplasia. *J Med Genet* **28**: 411–412

Huang A, Campbell CE, Bonetta L, McAndrews Hill MS, Chilton MacNeill S, Coppes MJ, Law DJ, Feinberg AP, Yeger H and Williams BR (1990) Tissue, developmental, and tumor-specific expression of divergent transcripts in Wilms' tumor. *Science* **250**: 991–994.

 © 2000 Cancer Research Campaign

Huff V and Saunders GF (1993) Wilms' tumor genes. *Biochim Biophys Acta* **1155**: 295–306
Kaneko Y, Homma C, Maseki N, Sakurai M and Hata J (1991) Correlation of chromosome abnormalities with histological and clinical features in Wilms' and other childhood renal tumors. *Cancer Res* **51**: 5937–5942
Keen TJ, Inglehearn CF, Green ED, Cunningham AF, Patel RJ, Peacock RE, Gerken S, White R, Wessenbach J and Bhattacharya SS (1995) YAC contig spanning the dominant retinitis pigmentosa locus (RP9) on chromosome 7p. *Genomics* **28**: 383–388
Knudson AG and Strong LC (1972) Mutation and cancer: a model for Wilms' tumour of the kidney. *J Natl Cancer Inst* **48**: 313–324
Lahoti C, Thorner P, Malkin D and Yeger H (1996) Immunohistochemical detection of p53 in Wilms' tumors correlates with unfavorable outcome. *Am J Pathol* **148**: 1577–1589
Lobbert RW, Klemm G, Gruttner HP, Harms D, Winterpacht A and Zabel BU (1998) Novel WT1 mutation, 11p LOH, and t(7;12) (p22;q22) chromosomal translocation identified in a Wilms' tumor case. *Genes Chromosomes Cancer* **21**: 347–350
McDonald JM, Douglass EC, Fisher R, Geiser CF, Krill CE, Strong LC, Virshup D and Huff V (1998) Linkage of familial Wilms' tumor predisposition to chromosome 19 and a two-locus model for the etiology of familial tumors. *Cancer Res* **58**: 1387–1390
Maitland NJ, Cox MF, Lynas C, Prime SS, Meanwell CA and Scully C (1987) Detection of human papillomavirus DNA in biopsies of human oral tissue. *Br J Cancer* **56**: 245–250
Malkin D, Sexsmith E, Yeger H, Williams BRG and Coppes MJ (1994) Mutations of the p53 tumor suppressor gene occur infrequently in Wilms' tumor. *Cancer Res* **54**: 2077–2079
Maw MA, Grundy PE, Millow LJ, Eccles MR, Dunn RS, Smith PJ, Feinberg AP, Law DJ, Paterson MC, Telzerow PE, Callen DF, Thompson AD, Richards RI and Reeve AE (1992) A 3rd Wilms' tumor locus on chromosome-16q. *Cancer Res* **52**: 3094–3098
Mellersh C and Sampson J (1993) Simplifying the detection of microsatellite length polymorphisms. *Biotechniques* **15**: 582–584
Miozzo M, Perotti D, Minoletti F, Mondini P, Pilotti S, Luksch R, Fossatibellani F, Pierotti MA, Sozzi G and Radice P (1996) Mapping of a putative tumor suppressor locus to proximal 7p in Wilms' tumors. *Genomics* **37**: 310–315
Miyagawa K, Kent J, Moore A, Charlieu J, Little M, Williamson KA, Kelsey A, Brown KW, Hassam S, Briner J, Hayashi Y, Hirai H, Yazaki Y, van Heyningen V and Hastie ND (1998) Loss of WT1 function leads to ectopic myogenesis in Wilms' tumor. *Nat Genet* **18**: 15–17
Ogawa O, Eccles MR, Szeto J, Mcnoe LA, Yun K, Maw MA, Smith PJ and Reeve AE (1993) Relaxation of insulin-like growth factor-II gene imprinting implicated in Wilms' tumor. *Nature* **362**: 749–751
Park S, Bernard A, Bove KE, Sens DA, Hazen-Martin DJ, Garvin AJ and Haber DA (1993) Inactivation of WT1 in nephrogenic rests, genetic precursors to Wilms' tumour. *Nat Genet* **5**: 363–367
Peier AM, Meloni AM, Erling MA and Sandberg AA (1995) Involvement of chromosome 7 in Wilms' tumor. *Cancer Genet Cytogenet* **79**: 92–94
Ping AJ, Reeve AE, Law DJ, Young MR, Boehnke M and Feinberg AP (1989) Genetic linkage of Beckwith–Wiedemann syndrome to 11p15. *Am J Hum Genet* **44**: 720–723
Radice P, Perotti D, Debenedetti V, Mondini P, Radice MT, Pilotti S, Luksch R, Bellani FF and Pierotti MA (1995) Allelotyping in Wilms' tumors identifies a putative third tumor suppressor gene on chromosome 11. *Genomics* **27**: 497–501
Rahman N, Arbour L, Tonin P, Renshaw J, Pelletier J, Baruchel S, Pritchardjones K, Stratton MR and Narod SA (1996) Evidence for a familial Wilms' tumour gene (FWT1) on chromosome 17q12–q21. *Nat Genet* **13**: 461–463
Rainier S, Johnson LA, Dobry CJ, Ping AJ, Grundy PE and Feinberg AP (1993) Relaxation of imprinted genes in human cancer. *Nature* **362**: 747–749
Reeve AE, Sih SA, Raizis AM and Feinberg AP (1989) Loss of allelic heterozygosity at a second locus on chromosome 11 in sporadic Wilms' tumor cells. *Mol Cell Biol* **9**: 1799–1803
Reik W and Maher ER (1997) Imprinting in clusters: lessons from Beckwith-Wiedemann syndrome. *Trend Genet* **13**: 330–334
Reynolds PA, Powlesland RM, Keen TJ, Inglehearn CF, Cunningham AF, Green ED and Brown KW (1996) Localization of a novel t(1:7) translocation associated with Wilms' tumor predisposition and skeletal abnormalities. *Genes Chromosomes Cancer* **17**: 151–155
Rivera H (1995) Constitutional and acquired rearrangements of chromosome 7 in Wilms' tumor. *Cancer Genet Cytogenet* **81**: 97–98
Rivera H, Ruiz C, Garcia Cruz D, Rolon A, Arroyo J and Cantu JM (1985) Constitutional mosaic t(2;7)(q33;p22) and other rearrangements in a girl with Wilms' tumor. *Ann Genet* **28**: 52–54
Sawyer JR, Winkel EW, Redman JF and Roloson GJ (1993) Translocation (7;7)(p13;q21) in a Wilms' tumor. *Cancer Genet Cytogenet* **69**: 57–59
Sheffield VC, Weber JL, Buetow KH, Murray JC, Even DA, Wiles K, Gastier JM, Pulido JC, Yandava C, Sunden SL, Mattes G, Businga T, Mcclain A, Beck J, Scherpier T, Gilliam J, Zhong J and Duyk GM (1995) A collection of tri- and tetranucleotide repeat markers used to generate high quality, high resolution human genome-wide linkage maps. *Hum Mol Genet* **4**: 1837–1844
Solis V, Pritchard J and Cowell JK (1988) Cytogenetic changes in Wilms' tumors. *Cancer Genet Cytogenet* **34**: 223–234
Steenman M, Redeker B, De Meulemeester M, Wiesmeijer K, Voute PA, Westerveld A, Slater R and Mannens M (1997) Comparative genomic hybridization analysis of Wilms' tumors. *Cytogenet Cell Genet* **77**: 296–303
Wang-Wuu S, Soukup S, Bove B, Gotwals B and Lampkin B (1990) Chromosomal analysis of 31 Wilms' tumors. *Cancer Res* **50**: 2786–2793
Weber JL, Kwitek AE and May PE (1990) Dinucleotide repeat polymorphisms at the D16S260, D16S261, D16S265, D16S266, and D16S267 loci. *Nucleic Acids Res* **18**: 4034
Wilmore HP, White GFJ, Howell RT and Brown KW (1994) Germline and somatic abnormalities of chromosome 7 in Wilms' tumor. *Cancer Genet Cytogenet* **77**: 93–98

© 2000 Cancer Research Campaign
